# Preparation and Toxicological Assessment of Functionalized Carbon Nanotube-Polymer Hybrids

**DOI:** 10.1371/journal.pone.0107029

**Published:** 2014-09-17

**Authors:** Nikos D. Koromilas, Georgia Ch. Lainioti, Chrisostomi Gialeli, Despoina Barbouri, Katerina B. Kouravelou, Nikos K. Karamanos, George A. Voyiatzis, Joannis K. Kallitsis

**Affiliations:** 1 Department of Chemistry, University of Patras, Patras, Greece; 2 Foundation for Research and Technology-Hellas (FORTH) / Institute of Chemical Engineering Sciences (ICE-HT), Rio-Patras, Greece; 3 Nanothinx S.A., Rio-Patras, Greece; Indian Institute of Toxicology Reserach, India

## Abstract

Novel Carbon Nanotube-Polymer Hybrids were synthesized as potential materials for the development of membranes for water treatment applications in the field of Membrane Bioreactors (MBRs). Due to the toxicological concerns regarding the use of nanomaterials in water treatment as well as the rising demand for safe drinking water to protect public health, we studied the functionalization of MWCNTs and Thin-MWCNTs as to control their properties and increase their ability of embedment into porous anisotropic polymeric membranes. Following the growth of the hydrophilic monomer on the surface of the properly functionalized CNTs, that act as initiator for the controlled radical polymerization (ATRP) of sodium styrene sulfonate (SSNa), the antimicrobial quaternized phosphonium and ammonium salts were attached on *CNTs-g-PSSNa* through non-covalent bonding. In another approach the covalent attachment of quaternized ammonium polymeric moieties of acrylic acid-vinyl benzyl chloride copolymers with N,N-dimethylhexadecylamine (P(AA12-co-VBCHAM)) on functionalized CNTs has also been attempted. Finally, the toxicological assessment in terms of cell viability and cell morphological changes revealed that surface characteristics play a major role in the biological response of functionalized CNTs.

## Introduction

In recent years, carbon nanotubes (CNTs) have been synthesized and mass-produced because of their unique optical, electrical, and mechanical properties leading to a broad range of application areas from consumer goods to medical applications [1]–[4].

However, their excellent properties are many times restricted by dispersion difficulties and low processability, such as the formation of aggregates due to Van der Waals interactions. Separation of these aggregates, as well as dispersion and alignment of CNTs are very important steps for many applications. Among several approaches to align nanotubes, surface modification or functionalization of CNTs, have been extensively explored, since the prevention of bundling will promote the homogenous dispersion and improve the miscibility of CNTs with matrices [5], [6]. Chemical modification, by covalent attachment and non-covalent adsorption or wrapping of functional polymers onto the CNT surfaces [7]–[11], has been proposed in order to improve the dispersion of CNTs in the polymer matrix [12].

CNTs are concentric cylinders of one or several graphene sheet(s) (single- or multi-wall carbon nanotubes respectively – SWCNTs and MWCNTs). Like other nanomaterials, their nanoscale dimensions may inflict potential adverse effects on human health, raising great concern among toxicologists and environmental scientists. Their unique physicochemical characteristics like length, diameter, number of sheets, structural defects, surface area, tendency to agglomerate, dispersibility in solution, presence and nature of catalyst residues, as well as surface chemistry, determine the reported biological reactivity and toxicity of CNTs [13]–[16]. More specifically, a large number of *in vitro* studies have been reported throughout the literature, demonstrating diverse effects of CNTs. For example, CNTs induced both cytotoxic and non-cytotoxic responses in the level of cell viability, production of free radicals, inflammation, cell morphology, DNA damage. Such diverse results depict the various experimental assays used, the material's properties, as well as cell type dependent effects [17]–[19]. In this line, the understanding of the toxicological impact of CNTs is a critical issue for the future application of these promising nanomaterials.

In this work, we use covalent functionalization of CNTs in order to control their properties but also their ability to be fixed onto porous membranes. Two types of CNTs, namely multiwalled (MWCNTs) or Thin-multiwalled (Thin-MWCNTs) carbon nanotubes were used in order to control the diameter of the final hybrid. The hydrophilic monomer sodium styrene sulfonate (SSNa) has been polymerized on properly modified CNTs using Atom Transfer Radical Polymerization (ATRP). In a further step, ammonium and phosphonium salts were introduced, through ionic interactions, in the PSSNa containing hybrids. Moreover, copolymers containing quaternized ammonium species covalently bounded were also used for the modification of CNTs. Quaternary ammonium and phosphonium groups are promising candidates for the development of antimicrobial agents. We applied two different experimental approaches to assess the pristine CNTs as well as the modified derivatives toxicological impact. Cell viability of normal lung fibroblasts was evaluated upon treatment with the materials. At a second step, we proceeded to cell morphological evaluations by immunofluorescence staining of lung cancer cells microtubules.

## Experimental Section

### Materials

Multi-walled carbon nanotubes of 97% purity as-produced (outer diameter of 15–35 nm) and Thin-Multi-walled carbon nanotubes of 94% purity as-produced (outer diameter of 6–15 nm) were produced using the Catalytic Chemical Vapor Deposition (CCVD) over mixtures of metal oxides, as the catalytic substrate (patented proprietary of Nanothinx S.A.), and hydrocarbons, as the carbon precursor, at a temperature range between 600 and 700°C. The monomers 4-vinylbenzyl chloride (VBC), sodium styrene sulfonate (SSNa), acrylic acid (AA), the initiator azobisisobutyronitrile (AIBN), the catalyst copper bromide (I) (CuBr, 99,999%), the ligands 2,2′-bipyridine (bipy), N,N,N′,N″,N″-Pentamethyldiethylenetriamine (PMDETA, 99%), the reagents 4-aminophenol, diethyl azodicarboxylate (DEAD), N,N-dimethylhexadecylamine, distilled triethylamine (Et_3_N), the dried solvent N,N-dimethylformamide (DMF_d_) are products of Aldrich. The solvents N,N-dimethylformamide (DMF), tetrahydrofuran (THF), diethyl ether, dichloromethane (DCM), chloroform (CHCl_3_), 1,2-dichloroethane (DCE) and hexane were purchased from Fischer. The reagents isopentyl nitrite and hexadecyltributylphosphonium bromide are products of Fluka and the reagents 2-chloropropionyl chloride (CPC) and triphenylphosphine (TPP), as well as the solvent acetone were purchased from Merck. The reagent hexadecyltrimethylammonium bromide was purchased from Acros Organics. Ultra-pure water was obtained by means of a SG apparatus water purification unit.

### Instruments and Measurements

Thermogravimetric analysis (TGA) was carried out in alumina crucibles in a Labsys TG apparatus of Setaram under nitrogen and at a heating rate of 10°C/min. The functionalization degree of carbon nanotubes was estimated according to: (% Carbon/atomic weight of carbon) / (% functional group/molecular weight of the functional group).

SEM-EDS experiments were performed on a Zeiss SUPRA 35VP instrument equipped with an EDS detector.

^1^H-NMR spectra were obtained on a Bruker Advance DPX 400 spectrometer, with CDCl_3_ as solvent containing TMS internal standard.

ATR-FTIR spectra were recorded on a Platinum. ATR Bruker.

Ultrasonic processor UP400S (400 watts, 24 kHz) by Heilscher Ultrasound Technology was utilized for the dispersion of CNTs using the following operating conditions Cycle 1, Amplitude 35%.

### Functionalization of carbon nanotubes

**Figure 1:**
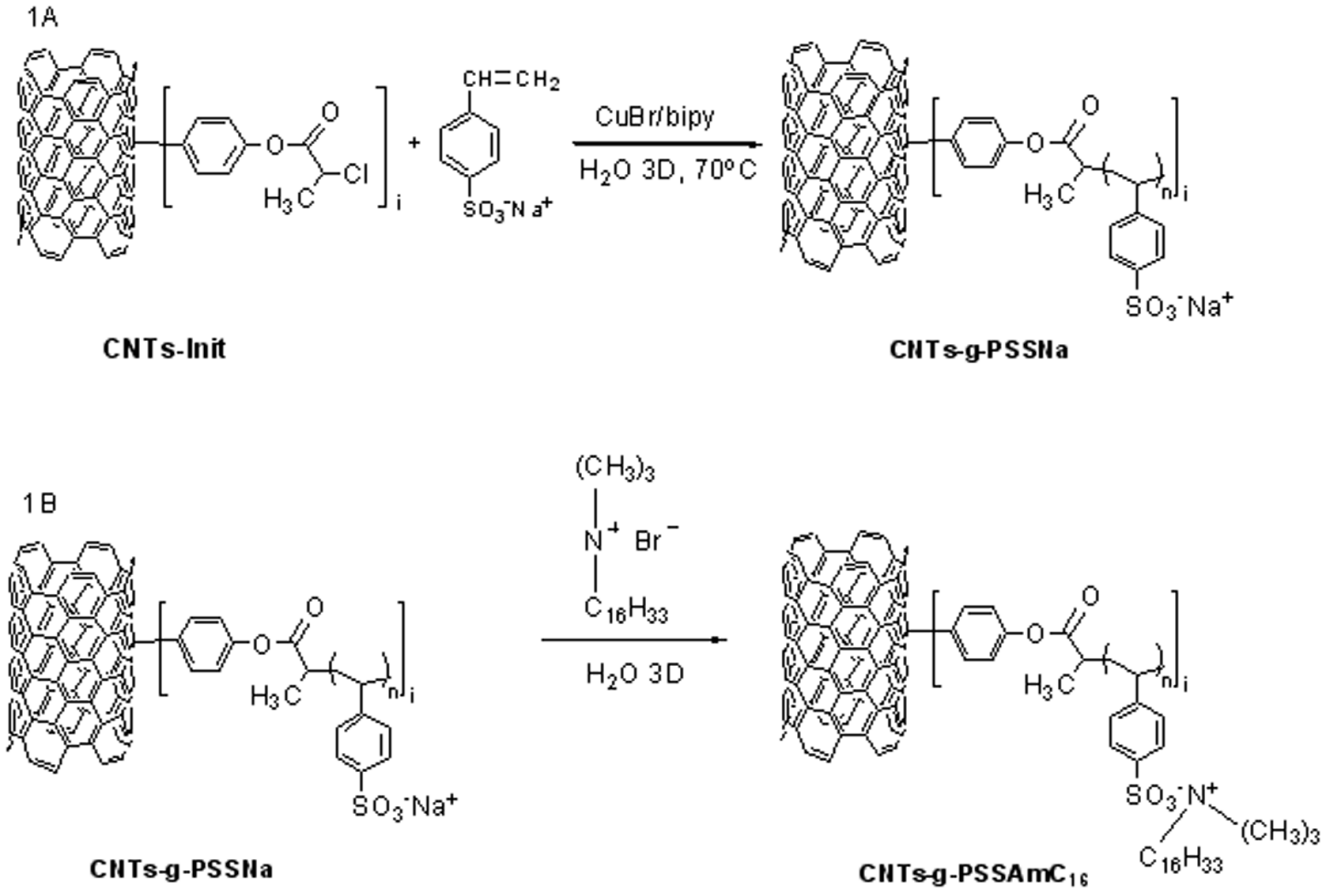
Schematic illustration of the functionalization of CNTs with SSNa moieties (A) and the introduction of quaternized ammonium groups (B).

MWCNTs and Thin-MWCNTs were initially functionalized with hydroxyl groups via the diazonium chemistry, as reported elsewhere [20], [21], using 4-aminophenol and isopentyl nitrite. In a typical reaction, 3.5 g of MWCNTs or Thin-MWCNTs were dispersed in 125 mL of triple distilled water (2.8 g/mL) after treatment in an ultrasonic bath for 30 minutes. Then, 8.3 g (76 mmol) of 4-aminophenol and 51 ml (380 mmol) isopentyl nitrite were added. The flask was placed at 60°C for 72 h. The resulting mixture (***MWCNTs-OH*** or ***Thin-MWCNTs-OH***) was vacuum filtered through Nylon membrane (200 nm), washed several times with triple distilled water, DMF and acetone, and dried under vacuum at 60°C over night. In a further step, the CNTs-OH were esterified with 2-chloropropionyl chloride for the attachment of initiator groups on the surface of nanotubes. In a degassed 150 mL round bottom flask, 0.5 g of MWCNTs or Thin-MWCNTs were dispersed in 100 mL of DMFd (0.5 g/mL) after treatment in an ultrasonic bath for 30 minutes. The mixture was placed in an ice bath and 20 ml of distilled triethylamine (Et_3_N) and 40 ml (400 mmol) of 2-chloropropionyl chloride (CPC) were added. The solution was degassed three times using vacuum and argon purging. The reaction was left to proceed at 80°C for 72 h. The product (***MWCNTs-Init*** or ***Thin-MWCNTs-Init***) was vacuum filtered through Nylon membrane (200 nm), washed several times with DMF, DCE and CHCl_3_, and dried under vacuum at 60°C over night. Furthermore, surface-initiated ATRP was employed for the polymerization of hydrophilic sodium styrene sulfonate (SSNa) monomer onto modified CNTs [22], as shown in **Figure 1A**. In a degassed 50 mL round bottom flask, 100 mg of MWCNTs-Init or Thin-MWCNTs-Init were dispersed in 25 mL of triple distilled water (0.4 g/mL). Next, 19.2 mg CuBr (0.13 mmol) and 21.5 mg (0.15 mmol) bipy were added and the flask was placed in an ultrasonic bath for 30 minutes. 3.09 g (15 mmol) of SSNa monomer was finally added. The solution was purged by three argon/vacuum cycles and was left at 80°C for 72 h. The obtained material (***MWCNTs-g-PSSNa*** or ***Thin-MWCNTs-g-PSSNa***) was vacuum filtered through Nylon membrane (200 nm), washed several times with triple distilled water, and dried under vacuum at 60°C over night. Functionalization procedures were confirmed with thermogravimetric analysis and the results are presented in **Table 1**.

**Table 1:** Functionalization of CNTs with ATRP initiator groups.

Material	TGA weight loss^1^ (%) at 700°C	Number of modified carbon atoms to Thin-MWCNTs per 1000 C
MWCNTs-OH	6	8
Thin-MWCNTs-OH	11	15
MWCNTs-Init	2	6
Thin-MWCNTs-Init	3	9

1 *compared to pristine CNTs.*

### Synthesis of quaternary ammonium and phosphonium-carbon nanotube hybrid nanomaterials

In a 100 mL round bottom flask, equipped with a magnetic stirrer, 30 mg of MWCNTs-g-PSSNa or Thin-MWCNTs-g-PSSNa (1.5 mg/mL) were dispersed in 20 mL of triple distilled water after treatment in an ultrasonic bath for 30 minutes. For the preparation of quaternized ammonium salt carbon nanotube hybrid nanomaterials, 500 mg of hexadecyltrimethylammonium bromide were added in the flask containing either MWCNTs-g-PSSNa or Thin-MWCNTs-g-PSSNa, as shown in **Figure 1B**. In the case of phosphonium-carbon nanotube hybrid nanomaterials, 150 mg of hexadecyltributylphosphonium bromide were added in the flask containing Thin-MWCNTs-g-PSSNa. In all cases, the flask was sealed with a glass plug and the reaction mixture was stirred at room temperature for 24 h. The next day, the resulting mixture was vacuum filtered through Nylon membrane (200 nm). The obtained product for each reaction (***MWCNTs-g-PSSAmC_16_, Thin-MWCNTs-g-PSSAmC_16_, Thin-MWCNTs-g-PSSPhC_16_***) was washed several times with triple distilled water and dried under vacuum at 70°C over night.

### Attachment of quaternized ammonium groups containing copolymer P(AA12-co-VBCHAM) onto CNT-OH

The synthesis of acrylic acid-vinyl benzyl chloride copolymers (P(AA12-co-VBC)) took place through free radical polymerization, followed by the quaternization of VBC units with N,N-dimethylhexadecylamine (HAM). Briefly, the desired quantity of the two monomers were placed in a 100 mL round bottom flask, equipped with a reflux condenser, and dissolved in chloroform. The solution was degassed, and the initiator AIBN (0.02 mol% over the total monomer concentration) was added. The reaction was left to proceed overnight under vigorous stirring in an Ar atmosphere in an oil bath set at 70°C. After cooling down to room temperature, the copolymers were recovered by precipitation in acetone, filtered washed and dried in a vacuum oven at 80°C for 24 h. Following this procedure, in a 100 mL round bottom flask, equipped with a magnetic stirrer, 1.5 g (9.2 mmol) of P(AA12-co-VBC) were dissolved in 50 mL of CHCl_3_ and 8.6 mL (25.5 mmol) of HAM were added. The flask was sealed with a glass plug and the reaction mixture was heated at 60°C for 3 days. After cooling down to room temperature, the product was recovered after precipitation in acetone, washed with hexane and dried under vacuum at 60°C over night.

**Figure 2:**
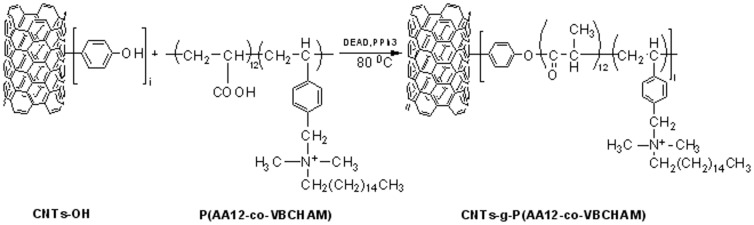
Schematic illustration of the functionalization of CNTs-OH with P(AA12-co-VBCHAM).

The quaternized copolymer was finally attached to the surface of hydroxyl-modified nanotubes through esterification reaction, as shown in **Figure 2**. Thus, CNTs-OH (100 mg), copolymer P(AA12-co-VBCHAM) (100 mg, 0.04 mmol), and triphenyl phosphine, TPP (25 mg, 0.095 mmol) were combined in a previously degassed round bottomed flask. DMF (10 mL) and THF (10 mL) were added and the reaction mixture was sonicated for 30 min. Diethyl azodicarboxylate (DEAD) (15 µL, 0.095 mmol) was then added and the flask was degassed and filled with argon several times. The reaction mixture was stirred at 80°C for 24 h under argon atmosphere. The same amounts of TPP and DEAD were then added to the flask once more and the resulting mixture was allowed to react at 80°C for a further 24 h under argon atmosphere. After being cooled to room temperature, the resulting mixture was vacuum filtered through Nylon membrane (200 nm). The obtained solid was washed several times with DMF, THF, CHCl_3_ and acetone and dried under vacuum at 70°C over night.

## Toxicological assessment of CNTs

### Biochemicals and reagents

Dulbeco's minimal essential medium (DMEM), Fetal bovine serum (FBS), sodium pyruvate, L-glutamine, penicillin, streptomycin, amphotericin B and gentamycin were all obtained from Biochrom KG (Berlin, Germany). All other chemicals used were of the best commercially available grade.

### Dispersion of materials

For optimal dispersion, the biocompatible surfactant polyoxyethylene-polyoxypropylene block copolymer – Pluoronic-127 (PF-127) was utilized. Initially, PF127 was dissolved in high-grade water at a concentration of 1% (w/v) and 3–5 mg of CNTs was subsequently added, followed by 10 min tip sonication. The concentration of the resulting stock solution in each case was 100 µg/mL.

### Cell lines and cell culture conditions

Lung fibroblasts DLF were isolated from healthy donors and cultured as monolayers in DMEM supplemented with 10% FBS as previously described [23], [24]. Human lung adenocarcinoma A549 cells were grown in DMEM/Ham's F-12 nutrient mixture containing 5% FBS. A cocktail of antimicrobial agents (100 IU/mL penicillin, 100 mg/mL streptomycin, 10 mg/mL gentamicin sulfate and 2.5 mg/mL amphotericin B), 2 mM L-glutamine and 1 mM sodium pyruvate were also supplemented to all cell cultures medium. Cultures were maintained at 37°C in a humidified atmosphere of 5% (v/v) CO_2_ and 95% air.

### Cell proliferation

In order to evaluate the effects of CNTs on cell proliferation, cells were seeded in the presence of serum into 96-well plates at a density of 7.500 cells/well. Twenty-four hours after plating, new medium supplemented with CNTs was added. A range of concentrations of 0.125 µg/mL to 25 µg/mL was evaluated for all assayed CNTs. Dilutions from the stock solution of CNTs were made in culture medium. The percentage of fetal bovine serum (FBS) was stable in all cases. No significant reduction of the total protein, due to CNTs properties, was observed.

After 24 h incubation, WST-1 (water-soluble tetrazolium salt) was added at a ratio 1∶10. This assay is based on the reduction of WST-1 reagent by viable cells, producing a soluble formazan salt and its absorbance is measured at 450 nm (reference wavelength at 650 nm), as described previously [25], [26]. All results were compared to the internal control of the assay 1% PF-127. Cytotoxicity was expressed as percentage of fibroblasts cells viability normalized to control cells. Comparisons with 1% PF-127 treated cells – internal control of the assay were also conducted.

Nanomaterials are reported to interfere with cytotoxicity tests due to their unique physicochemical properties. For this purpose, internal controls of the assays were performed, by incubating dyes with carbon nanotubes alone, and evaluated to the final results [27], [28]. In order to demonstrate that cell proliferation assay is working in each case, cells were incubated with high concentration of DMSO (10% DMSO), known cytotoxic agent [29].

### Immunofluorescence Staining of Cellular Microtubules

Cells were seeded on glass coverslips in 24-well plates and grown to confluence prior to treatment. Cells were washed twice with PBS buffer, fixed in 4% formaldehyde in PBS buffer, washed three times with PBS-Tween buffer, permeabilized with freshly made 0.5% Triton X-100 in PBS, washed three times with PBS-Tween buffer and blocked with 5% BSA in PBS-Tween. Microtubules were visualized (×60 magnification) by immunofluorescence staining using a mouse monoclonal antibody against α-tubulin (1∶100; Sigma) and an Alexa Fluor 594 anti-mouse second antibody (1∶2000; Invitrogen). Then, the coverslips were mounted on microscope slides. Alterations in microtubule morphology were assessed by comparison of photo images of control and treated cells from three individual experiments.

## Results and Discussion

### Introduction of quaternized ammonium and phosphonium salts onto CNT-g-PSSNa through ionic interactions

The general procedure for the formation of carbon nanotube-polymer hybrid nanomaterials, included the following steps: introduction of hydroxyl groups to the surface of carbon nanotubes through diazonium chemistry, attachment of isopropyl chloride groups that can act as initiators for atom transfer radical polymerization [11], [30] onto hydroxyl-functionalized carbon nanotubes and grafting polymerization of monomers from the surface of initiator-functionalized carbon nanotubes. MWCNTs and Thin-MWCNTs were properly modified following the above-mentioned steps, for the polymerization, under ATRP conditions, of the sodium styrene sulfonate monomer. For the identification of functionalization, thermogravimetric analysis (TGA) was performed for all materials. According to TGA curves, efficient **hydroxyl** functionalization for MWCNTs and Thin-MWCNTs was obtained with 6% and 11% weight loss, respectively, corresponding to 8 and 15 phenol groups attached covalently onto every 1000 carbon atoms (**Table 1**). After the introduction of the **initiator** groups, TGA analysis showed an additional weight loss of 2% and 3% for MWCNTs and Thin-MWCNTs, corresponding to the 2-chloropropionyl group attached to the phenol groups, indicating the presence of 6 and 9 initiator groups attached onto every 1000 carbon atoms, respectively.

**Figure 3:**
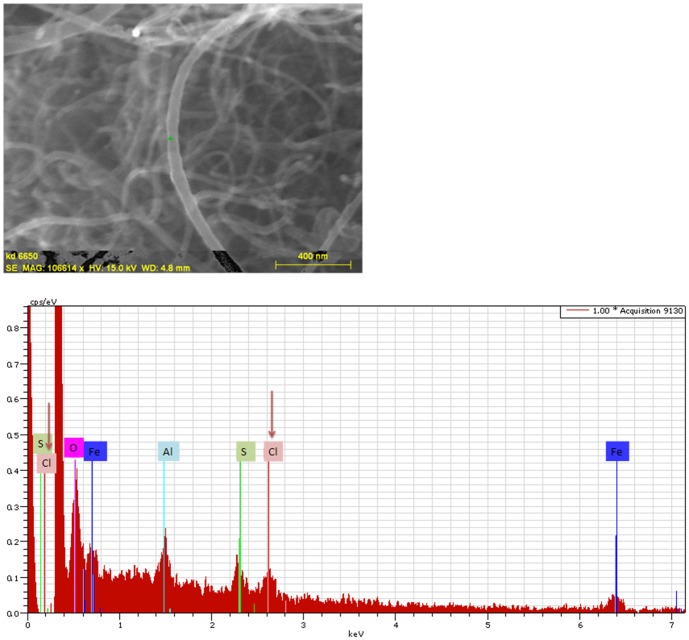
SEM-EDS analysis of Thin-MWCNTs-Init.

An additional process in order to confirm the modification of CNTs with initiator groups was the EDS analysis, which is one of the most powerful tools for identifying and quantifying (semi-quantitatively) the surface elements. Indicatively, **Figure 3** presents the scanning electron microscopy (SEM) image of Thin-MWCNTs-Init and the EDS spectra, which shows the appearance of chloride atom, a strong indicator of the introduction of 2-chloropropionyl chloride in Thin-MWCNTs-OH. Moreover, iron and aluminum atoms were detected, as well as sulfur atom, which were used as catalysts (for the synthesis of CNTs) and substrate, respectively.

The subsequent polymerization of sodium styrene sulfonate (**SSNa**) monomer onto the surface of CNTs-Init through ATRP, shown in **Figure 1A**, led to a 4% and 3.5% additional weight loss, compared to the MW and Thin-MWCNTs-Init. This indicated 3–6 and 2–4 monomers per initiator site for MWCNTs and Thin-MWCNTs respectively for 50–100% functionalization, as shown in **Table 2** [31].

**Table 2:** Polymerization of sodium styrene sulfonate (SSNa) onto the CNT-Init surface.

Material	Μol Init/mol CuBr/mol PMDETA/mol SSNa^1^	TGA weight loss^2^(%) at 700°C	TGA weight loss 3(%) at 700°C	Μ_n, TGA_ 4	Monomer units/initiator site (for 50%–100% fuctionalization)
MWCNTs-g-PSSNa	1/1.8/1.8/180	4	18	1802	3–6
Τhin MWCNTs-g-PSSNa	1/4/4/133	3.5	11	2650	2–4

1 mol of initiator  =  [(TGA weight loss of Thin-MWCNTs-Init - TGA weight loss of Thin-MWCNTs-ΟΗ)*mass of Thin-MWCNTs-Init]/(molecular weight of imported group*100).

2 *compared to CNTs-Init.*

3 *compared to PSSNa.*

4 Μn,TGA  =  theoretically TGA weight loss of Thin MWCNTs-g-PSSNa/[(1- theoretically TGA weight loss of Thin-MWCNTs-g-PSSNa)*(mol of initiator/g of initiator)].

**Figure 4:**
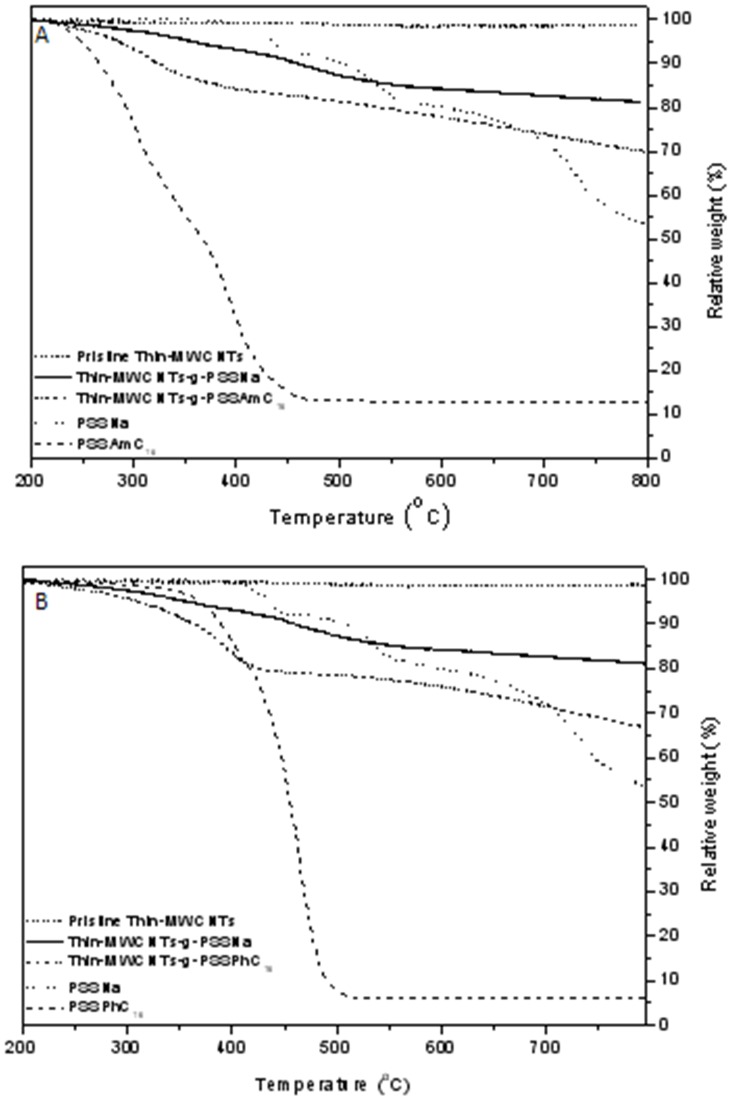
TGA curves of pristine Thin-MWCNTs and Thin-MWCNTs-g-PSSNa, as well as Τhin-MWCNTs-g-PSSAmC_16_ (A), and Τhin MWCNTs-g-PSSPhC_16_ (B)_._ TGA curves of the copolymers of PSSNa and PSSPhC_16_ are also shown.

In a further step, introduction of quaternized ammonium and phosphonium salts to CNTs-g-PSSNa took place, through ionic interactions with the sulfonic groups of the SSNa units. Quaternized groups were used for the functionalization of the CNT-polymer hybrid nanomaterials due to their potential biocidal activity. The antibacterial moieties in polymers have advantages such as non-volatilization, inability to permeate the skin and reduced toxicity to the environment, compared with conventional antibacterial agents of low molecular weight [32]. The schematic representation for the synthesis of quaternized ammonium- carbon nanotubes is shown in **Figure 1B** **.** The successful introduction of the quaternized groups was also confirmed through TGA analysis. As shown in **Figure 4A** the increased weight losses of 13% and 7% observed for MW and Thin-MWCNTs-g-PSSAmC_16_, respectively, lead to a 100% and 43% exchange after the introduction of ammonium salts (data for MWCNTs not shown). The degradation of CNTs-g-PSSAmC_16_ starts at approximately 200°C, which is consistent with the decomposition of quaternary ammonium salts [33]. However, the thermal stability of phosphonium salts is much higher, so the degradation of CNTs-g-PSSPhC_16_ starts at approximately 350°C (**Figure 4B**).

### Functionalization of CNTs with copolymers bearing quaternary ammonium groups covalently bounded

In order to introduce a covalent functionalization of CNTs with biocidal units, a copolymer bearing both the ammonium groups bounded on the polymer backbone and also carboxyl groups, that enable their further reaction with hydroxyl-functionalized CNTs, was synthesized. Thus, in the first stage, free radical polymerization technique was employed for the synthesis of acrylic acid-vinyl benzyl chloride copolymer (P(AA12-co-VBC)). Then, quaternization of VBC units occurred after addition of N,N-dimethylhexadecylamine. In the last stage, shown in **Figure 2**, introduction of P(AA12-co-VBCHAM) onto CNTs-OH was accomplished by an ester bond formation in the presence of triphenylphosphine (TPP) and diethyl azodicarboxylate (DEAD) (Mitsunobu-type reaction).

**Figure 5:**
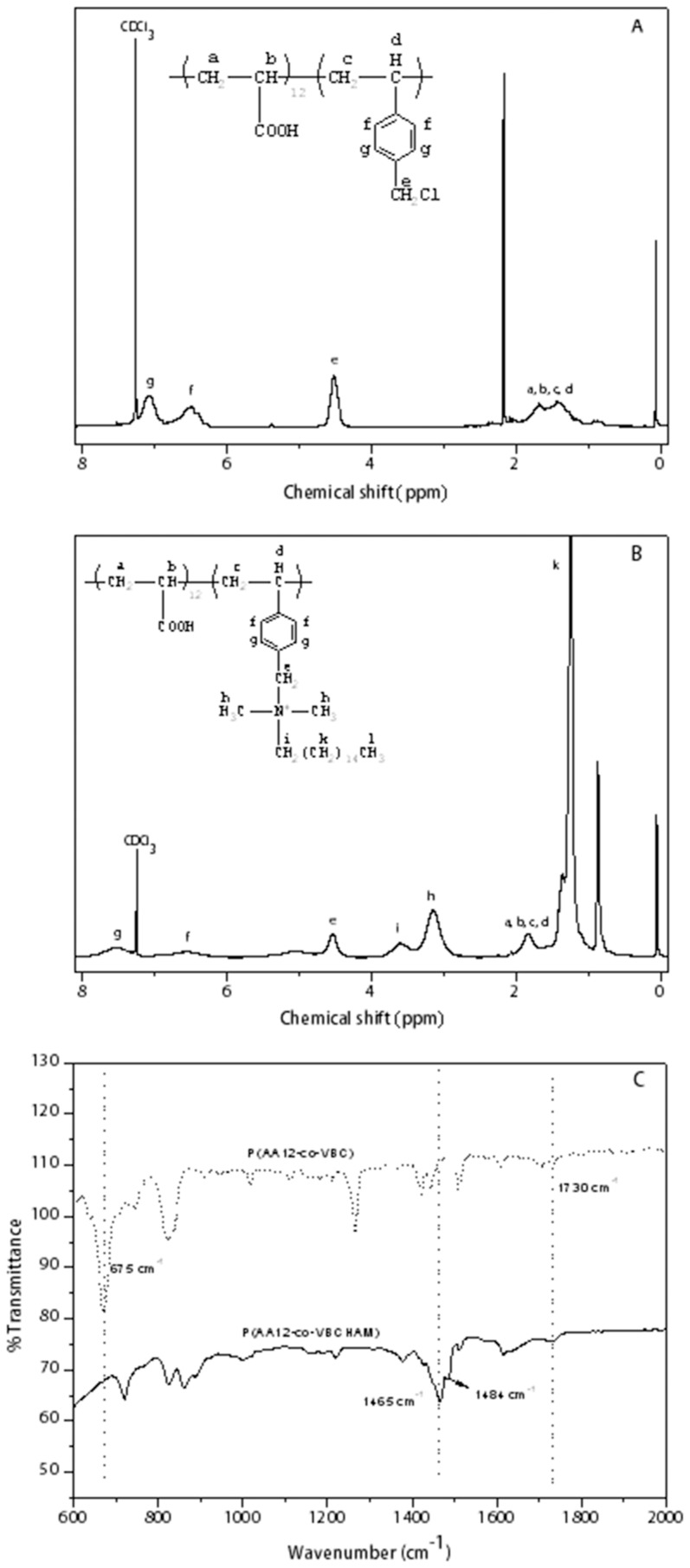
^1^Η-NMR spectra of the copolymer P(AA12-co-VBC) (A) and the quaternized copolymer P(AA12-co-VBCHAM) (B) and FT-IR spectra of the above copolymers (C).

The chemical structure of the statistical copolymers P(AA12-co-VBC) was determined by ^1^H-NMR, as shown in **Figure 5A**. VBC polymerization was confirmed also from the broad peaks at 4.5 ppm and at 6.2–7.1ppm, which correspond to the protons linked with the chloride atom (CH_2_Cl) and the aromatic peaks respectively. By quantification of ^1^Η-NMR, from the integration of CH_2_ and CH peaks at 1.4–1.7 ppm and CH_2_Cl peak at 4.5 ppm it is concluded that the percentage of acrylic acid in the copolymer is 12%.

The quaternization of the chloromethyl group was performed after typical reaction with the corresponding amine and the results are shown in **Figure 5B**. The successful introduction of quaternary ammonium groups was confirmed by the appearance of four new peaks at 0.8 ppm, 1.2 ppm, 3.1 ppm and 3.6 ppm, as well as the shift of the aromatic peak (g) from 7.1 to 7.5 ppm. The protons of CH_3_ group were found at 0.8 ppm, whereas the CH_2_ groups were identified at 1.2ppm. The remaining two peaks at 3.1 and 3.6 ppm were observed due to CH_3_ and CH_2_ groups, respectively, linked with the nitrogen atom. By integrating the peaks at 0.8 ppm and 4.5 ppm, which correspond to the protons of CH_3_ group and the protons of CH_2_ group linked to the chloride atom, it was found that the quaternization was accomplished 100%.

**Figure 6:**
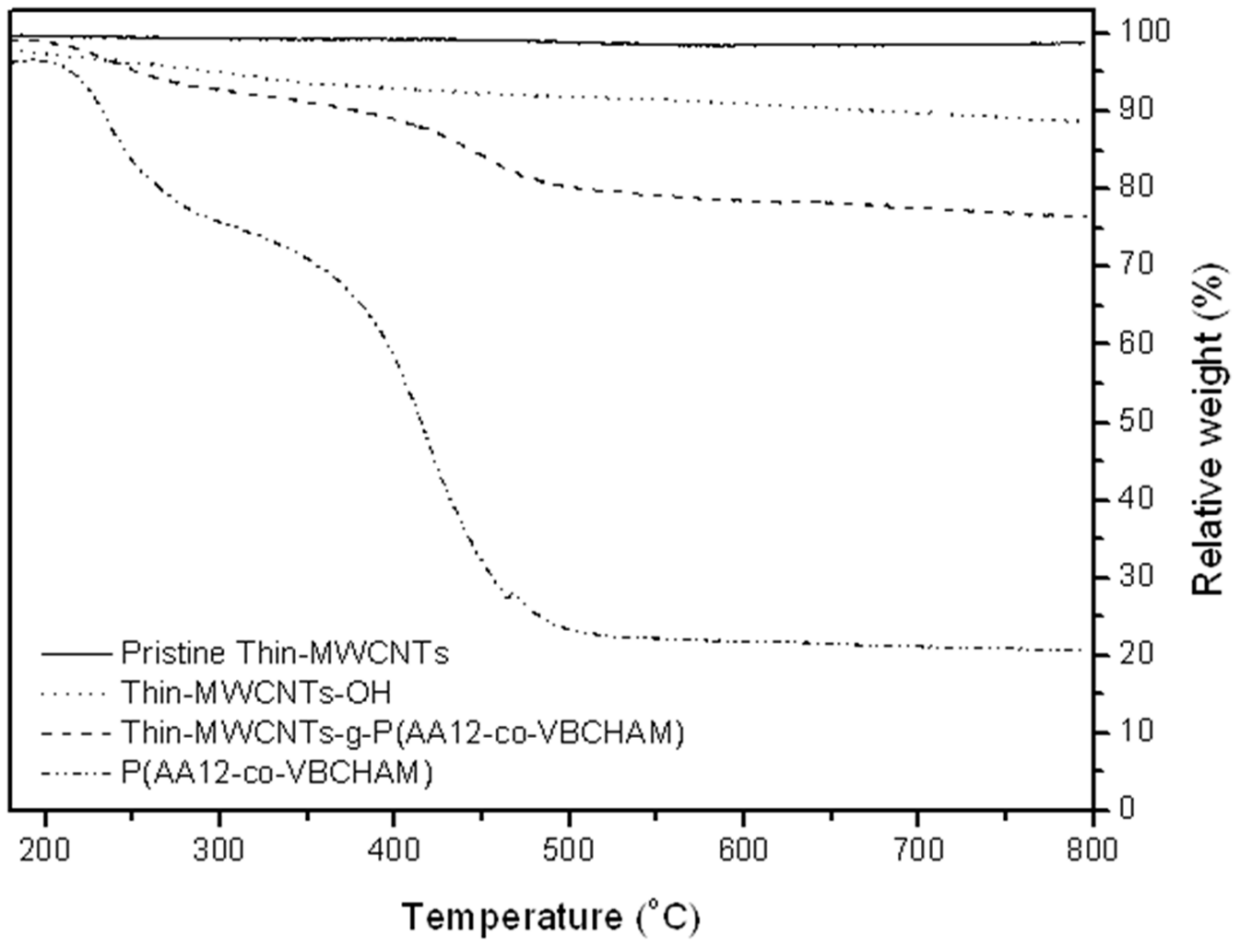
TGA curves of pristine Thin-MWCNTs, Thin-MWCNTs-ΟH, Thin-MWCNTs-g-P(ΑΑ12-co-VBCHAM) and the quaternized P(ΑΑ12-co-VBCHAM).

Additionally, FT-IR spectra of both the P(AA12-co-VBC) copolymer and the P(AA12-co-VBCHAM) quaternized copolymer are shown in **Figure 5C**. The C-Cl absorbance band appeared at 675 cm^−1^, revealing the VBC contribution [34], while the polymerization of PAA was confirmed by the carbonyl group at 1730 cm^−1^ [35]. After quaternization, the C-Cl peaks in the FT-IR spectra disappeared and two new peaks appeared at 1465 and 1484 cm^−1^ assigned to the CH_3_ bending of the amines and the C-N formation bond respectively [36]. In a further step, the functionalization of MWCNTs with the quaternized copolymer P(AA12-co-VBCHAM), was determined through thermogravimetric analysis. In the TGA, shown in **Figure 6**, the incorporation of the antimicrobial polymer on the CNTs-OH, leads to an increase in weight loss at the range of 12% for both MWCNTs (not shown here) and Thin-MWCNTs. In order to prove the polymer attachment on the surface of CNTs, the TGA thermogram of P(AA12-co-VBC) was also recorded, where the two degradation steps of the polymer are clearly shown.

### Toxicological assessment of CNTs

The use of *in vitro* models with endpoints that reveal a general mechanism of toxicity represent a promising basis for further assessment of the potential risk of exposure to nanomaterials in general [37].

An important factor of *in vitro* studies that contribute to the observed variations in CNT toxicity is the toxicological assessment method and the specific technical details of the experimental process, like CNT concentration, exposure time, and cell types used [38].

In the production line of membrane bioreactors the staff is exposed to high amounts of the synthesized hybrid and composite materials raising safety concerns. In this line, in the present study for the toxicological assessments we utilized cell lung models. The initial experiments performed in the present study were cell viability assays upon the exposure of the normal lung fibroblasts with increasing concentrations of pristine and synthesized/functionalized CNTs (range of concentrations from 0.125 µg/mL to 25 µg/mL). It is established in the literature that cancer cells, due to their high growth rates, are routinely utilized for cytotoxic evaluations in comparison with normal cells [39], [40]. So, in this work, we proceeded to cell morphological evaluations by immunofluorescence staining of lung cancer cells microtubules incubated with the upper and lower concentrations of cell viability assays. Microfilaments, microtubules and intermediate filaments are fundamental structures of the cytoskeleton. In the present study, the morphology of tubulin networks in cells was investigated by immunofluorescence staining using a specific antibody against α-tubulin. In this way, the observed tubulin modifications may be depicted on microtubule properties, such as stability and structure, as well as cellular events, such as cell division-proliferation and intracellular trafficking [41], [42].

### Evaluation of surfactant toxicity and pristine CNTs

The formation of CNTs aggregates may lead to increased CNT toxicity, due to altered CNTs-cells interactions [43]. In order to overcome the highly hydrophobic nature of CNTs and their immediate agglomeration in the culture media a number of approaches have been utilized that involve the use of surfactants. Indeed, there are several classes of surfactants - anionic, cationic, amphoteric and non-ionic – categorized by their polar functional groups. The ideal surfactant used to prevent aggregation in biological media is preferable due to high biocompatibility and low toxicity to the cells [44], [45].

**Figure 7:**
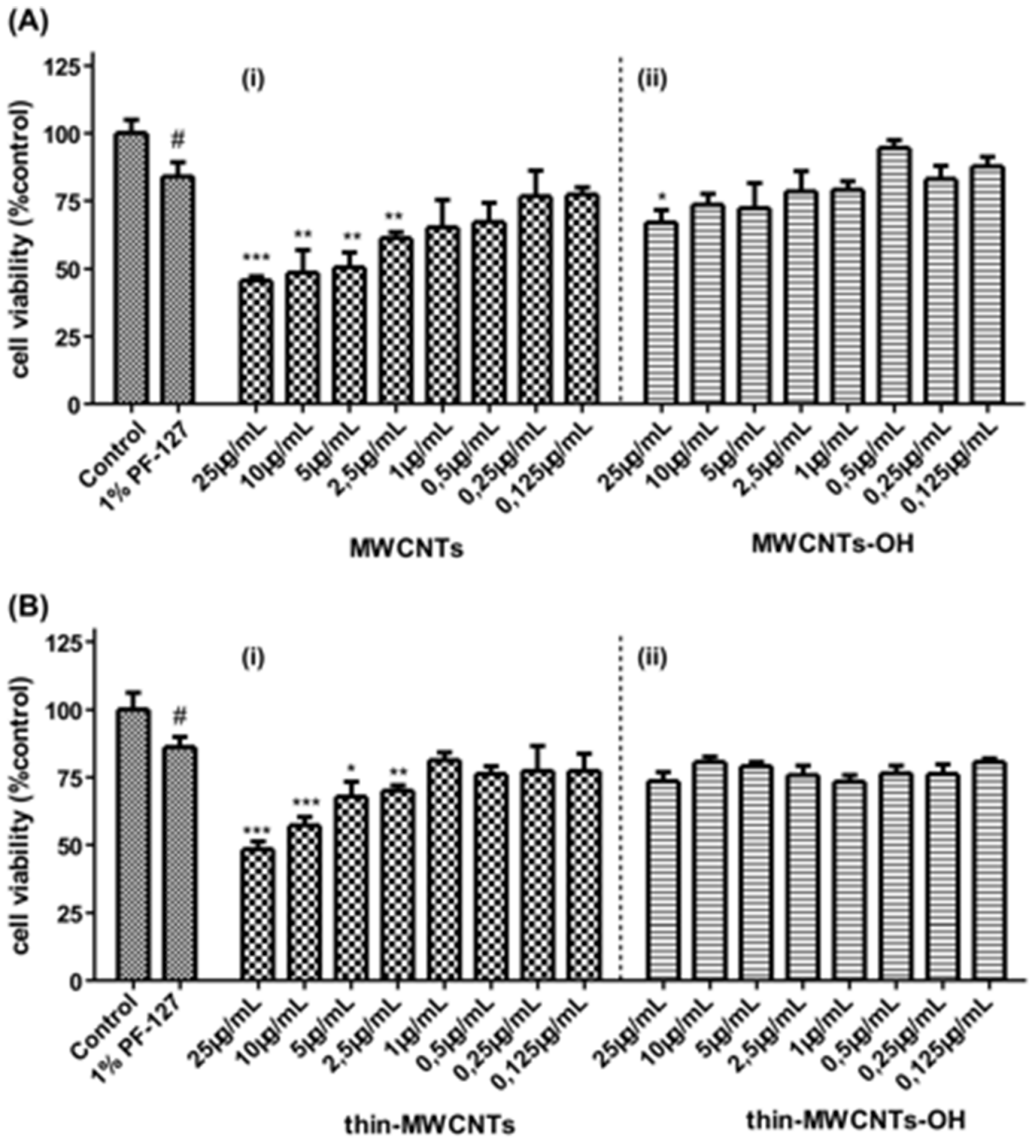
Effects of pristine and hydroxyl decorated CNTs on lung fibroblasts cells proliferation for a 24 h incubation period. (**A**) ***i*** *.* Pristine MWCNTs and ***ii*** *.* MWCNTs-OH and (**B**) ***i***. Pristine Thin-MWCNTs and ***ii*** *.* Thin-MWCNTs-OH. A range of concentrations from 0.125 µg/mL to 25 µg/mL was assayed. The results are expressed as mean ±SD of three separate experiments in triplicate. Statistically significant differences were evaluated using the ANOVA test. Statistically significant differences among the 1% PF-127 treated and control cells are shown by (#) (p≤0.05). Statistically significant differences among the CNTs-treated and 1% PF-127 are shown by (*) (p≤0.05), (**) (p≤0.01) and (***) (p≤0.001).

**Figure 8:**
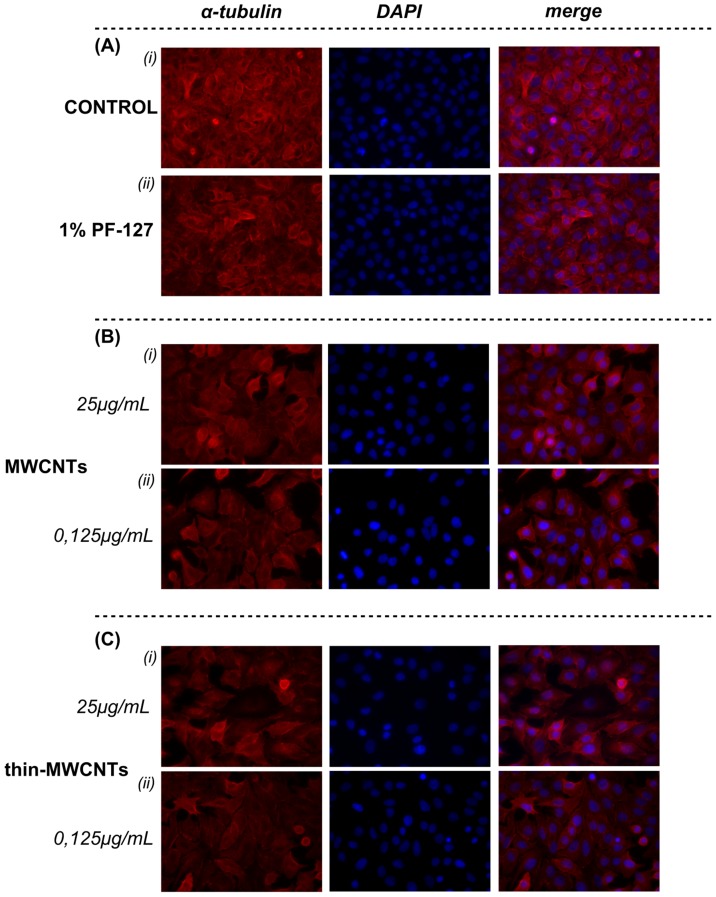
Effects of surfactant and pristine CNTs on lung cancer cells microtubules. Immunofluorescence staining was performed for α-tubulin (red) and nuclei (blue) in A549 cells after permeabilization. Data are representative of three independent experiments. Panel **A. (** ***i*** **)** Untreated Control cells and (***ii***) 1% PF-127. Panel **B.** MWCNTs (***i***) 25 µg/mL and (***ii***) 0.125 µg/mL. Panel **C**. Thin- MWCNTs (***i***) 25 µg/mL and (***ii***) 0.125 µg/mL.

In the present study, for optimal dispersion, we used the non-ionic surfactant polyoxyethylene-polyoxypropylene block copolymer– Pluoronic-127 (PF-127). It is worth mentioning that PF-127 concentration is critical as it has been reported that higher concentration might result in lower dispersion outcomes, further suggesting an intrinsic interaction between surfactant and CNTs [46]. To determine the optimal surfactant concentration, studies were conducted with lung fibroblasts. To note, the viability of cells exposed to the MWCNTs with no surfactant did not differ statistically from MWCNTs plus 1% PF-127 (data not shown). We observed a cell viability of 80–90% with the 1% PF-127 which was the internal control of all the experiments conducted; the effect of CNTs was always co-evaluated in comparison with 1% PF-127 treated cells, as the toxicity of the surfactant is included in the observed toxicity of CNTs (**Figure 7**). In addition, untreated lung cancer A549 cells possessed an overall solid cytoskeletal scaffold and formation of tubulin filaments (**Figure 8A** **i**). Microtubules extended uniformly throughout the cytoplasm and usually accumulated in the perinuclear region of control cells (**Figure 8** **Ai**). Mitotic cells were observed as well in several immunofluorescence images. Treatment of cells with 1% PF-127 resulted in no apparent changes in microtubule plasticity/dynamics (**Figure 8** **Aii**).

As far as pristine CNTs are concerned, by evaluating the percentage of viable cells we observed a dose-dependent effect on cell viability resulting in a 50–60% cell toxicity (**Figures 7** **Ai & 7 Bi**). In several *in vitro* studies in lung cell models, a dose-dependent, as well as a time-dependent effect was observed upon treatment with MWCNTs. Specifically, CNTs elicited a low acute cytotoxic effect during 24 h exposure with different kinds of CNTs, and this effect was at different extent depending on the experimental set-up [47]–[49]. Such outcomes are postulated to be mediated through an oxidative mechanism, involving perhaps production of reactive oxygen species, as well as an inflammatory response [38], [48].

A moderate disruption of microtubule network of A549 lung cancer cells is reported upon treatment with pristine CNTs, especially at the high concentration. Following exposure of the cells to CNTs, several enlarged cells with swollen nuclei were observed in **Figures 8B & 8C**. An increased number of apoptotic features, especially in the high concentration tested were also observed. More specifically, cells with bundles of microtubules around the condensed and/or fragmented nucleus were distinguished. Additionally, CNTs-treated A549 cells possessed an increased formation of microtubule filaments (**Figures 8B & 8C**). It is worth highlighting that the CNTs effects' on microtubules network are in accordance with the cell viability observations. In a recent study, immunofluorescence staining for F-actin of cytoskeleton revealed significant differences upon treatment with CNTs, forming dot- like or nodular-like structures in the border of the cell in comparison with thick stress fibers of control cells [49]. In the present study, no apparent changes on the induced cell toxicity were observed between the two types of pristine MWCNTs, utilized in terms of both cell viability and tubulin formation (**Figures 7** **&** **8B–C**). It is established in the literature that nanotube diameter is an important parameter to be considered in the toxicological assessment of CNTs. Moreover, it is noted that Thin-MWCNTs appeared significantly more toxic than the thicker ones, when dealing with 9.5 nm versus 70 nm diameter of CNTs [50]. In our case, the diameter of Thin-MWCNTs was 2-times lower compared with MWCNTs which may contribute to the non-apparent changes observed. However, in our set of experiments a non-significant trend is documented, depicting a slightly less toxic effect of Thin-MWCNTs compared to MWCNTs. On the other hand, hydroxyl-functionalization of CNTs resulted in maintenance of high viability profile even at high concentrations as compared to pristine CNTs (**Figures 7A** **ii & 7Bii**).

### Evaluation of SSNa decorated CNTs toxicity

It has been demonstrated that surface characteristics play a major role in the biological response of CNTs and that surface modifications by coating with polymers may result in decreased CNT toxicity, without affecting their intrinsic structure [15], [51].

**Figure 9:**
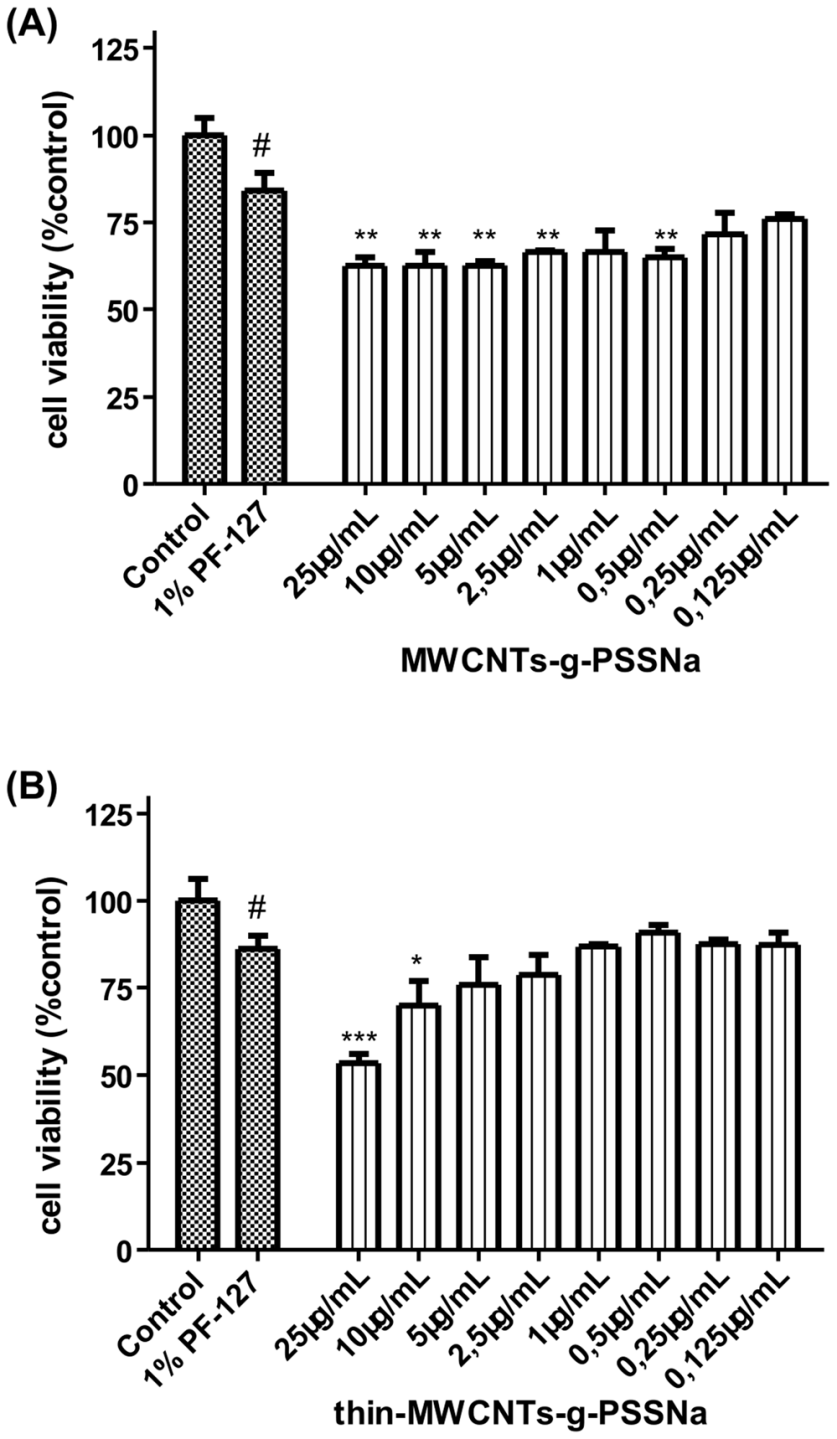
Effects of SSNa decorated CNTs on lung fibroblasts cells proliferation for a 24h incubation period. (**A**) MWCNTs-g-PSSNa and (**B**) Thin-MWCNTs-g-PSSNa. A range of concentrations from 0.125 µg/mL to 25 µg/mL was assayed. The results are expressed as mean ±SD of three separate experiments in triplicate. Statistically significant differences were evaluated using the ANOVA test. Statistically significant differences among the 1% PF-127 treated and control cells are shown by (#) (p≤0.05). Statistically significant differences among the CNTs-treated and 1% PF-127 are shown by (*) (p≤0.05), (**) (p≤0.01) and (***) (p≤0.001).

In the present study, CNTs functionalized with the water soluble polymer PSSNa and stabilized in aqueous media, resulted in especially high concentrations, in terms of viability (65–75%), compared to pristine CNTs (**Figures 7** **&** **9**). This difference is higher in MWCNTs compared to Thin-MWCNTs, probably due to the higher functionalization degree of PSSNa. Notably, a different localization of CNTs was observed with optical microscopy when they were functionalized with PSSNa – mainly pericellular – in contrast with the pristine that exhibited a more diffused pattern in the extracellular and pericellular space (data not shown). Similar pattern was also documented for Thin-MWCNTs with small deviations, as the characteristics of the CNTs are similar (**Figure 9**).

### Evaluation of PSSPhC_16_ and PSSAmC_16_ decorated CNTs toxicity

**Figure 10:**
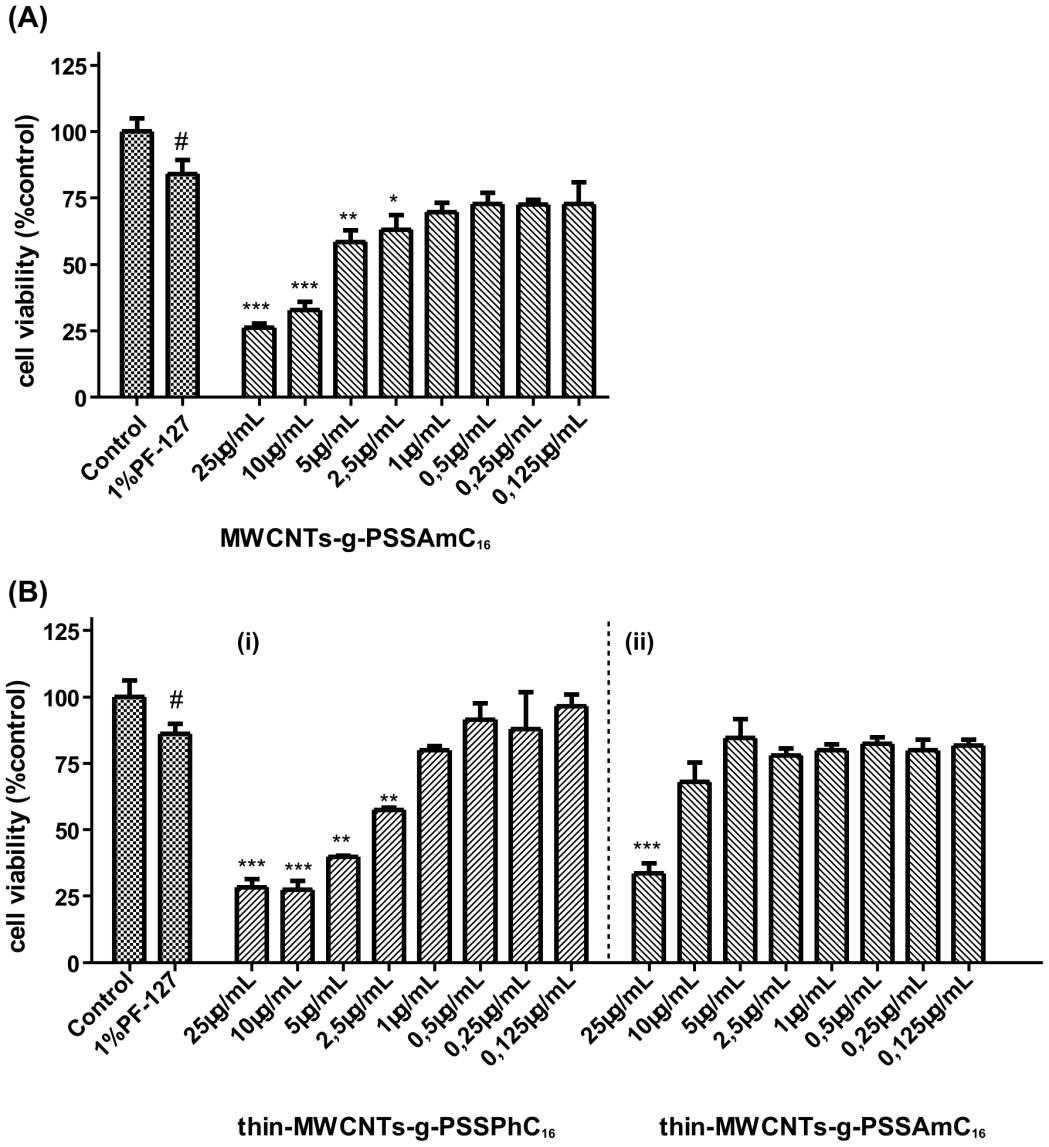
Effects of PSSPhC_16_ and PSSAmC_16_ decorated CNTs on lung fibroblasts cells proliferation for a 24 h incubation period. (**A**) MWCNTs-g-PSSAmC_16_ and (**B**) ***i*** *.* Thin-MWCNTs-g-PSSPhC_16_ and ***ii*** *.* Thin-MWCNTs-g-PSSAmC_16_. A range of concentrations from 0.125 µg/mL to 25 µg/mL was assayed. The results are expressed as mean ±SD of three separate experiments in triplicate. Statistically significant differences were evaluated using the ANOVA test. Statistically significant differences among the 1% PF-127 treated and control cells are shown by (#) (p≤0.05). Statistically significant differences among the CNTs-treated and 1% PF-127 are shown by (*) (p≤0.05), (**) (p≤0.01) and (***) (p≤0.001).

Non-covalent interaction of phosphonium and ammonium groups with SSNa-functionalized Thin-MWCNTs leads to high toxicity profile at concentrations higher than 2.5 µg/mL and 25 µg/mL, respectively (**Figures 10B** **i & ii**). On the other hand, non-covalent interaction of ammonium groups with SSNa-functionalized MWCNTs leads to high toxicity profile at concentrations higher than 5 µg/mL (**Figure 10A**). In both cases, at high concentrations of CNTs tested, cell toxicity approximately reached 75%. Such observations may be in part attributed to the antimicrobial nature of the decorated groups, as the CNTs functionalized with phosphonium groups showed higher capacity than ammonium-functionalized CNTs. However, in lower concentrations tested, cell viability reaches the levels of the internal control of the assay (**Figure 10**).

**Figure 11:**
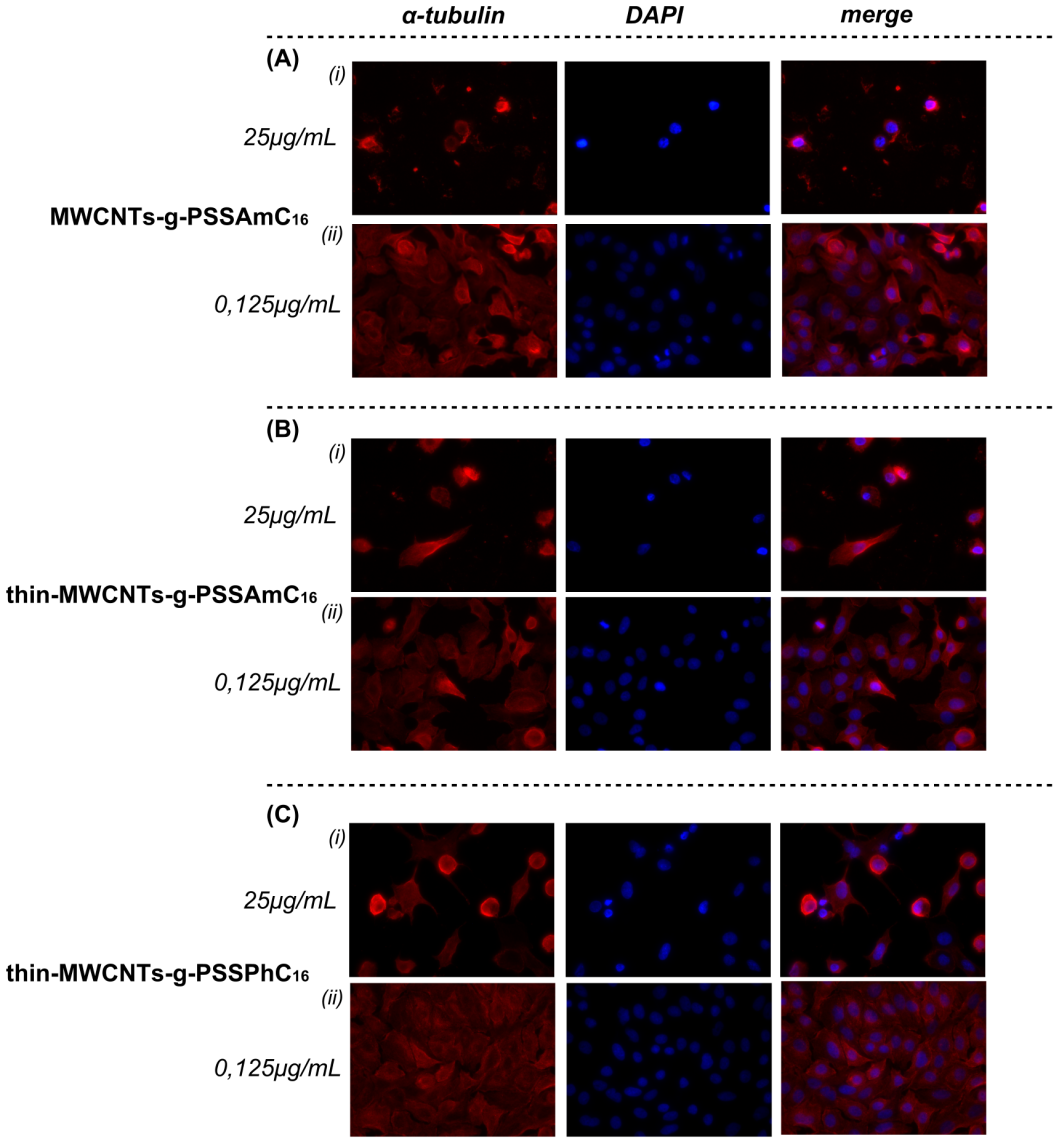
Effect of PSSPhC_16_ and PSSAmC_16_ decorated CNTs on lung cancer cells microtubules. Immunofluorescence staining was performed for α-tubulin (red) and nuclei (blue) in A549 cells after permeabilization. Data are representative of three independent experiments. Panel **A.** MWCNTs-g-PSSAmC_16_ (***i***) 25 µg/mL and (***ii***) 0.125 µg/mL. Panel **B**. Thin-MWCNTs-g-PSSAPhC_16_ (***i***) 25 µg/mL and (***ii***) 0.125 µg/mL. Panel **C.** Thin-MWCNTs-g-PSSAmC_16_ (***i***) 25 µg/mL and (***ii***) 0.125 µg/mL.

Gross morphological changes on A549 cells were observed in the experiments conducted with PSSPhC_16_ and PSSAmC_16_ decorated CNTs, especially at high concentrations (**Figure 11**). Results were in accordance with the high toxicity profile that was reported with the cell viability assay. Apart from the increased number of apoptotic features, in terms of cells with bundles of microtubules around the condensed and/or fragmented nucleus, PSSPhC_16_ and PSSAmC_16_ decorated CNTs treatments also caused the appearance of shrunken and rounded cells (**Figures 11A** **i, -Bi, -Ci**).

### Evaluation of P(AA12-co-VBCHAM) decorated CNTs toxicity

**Figure 12:**
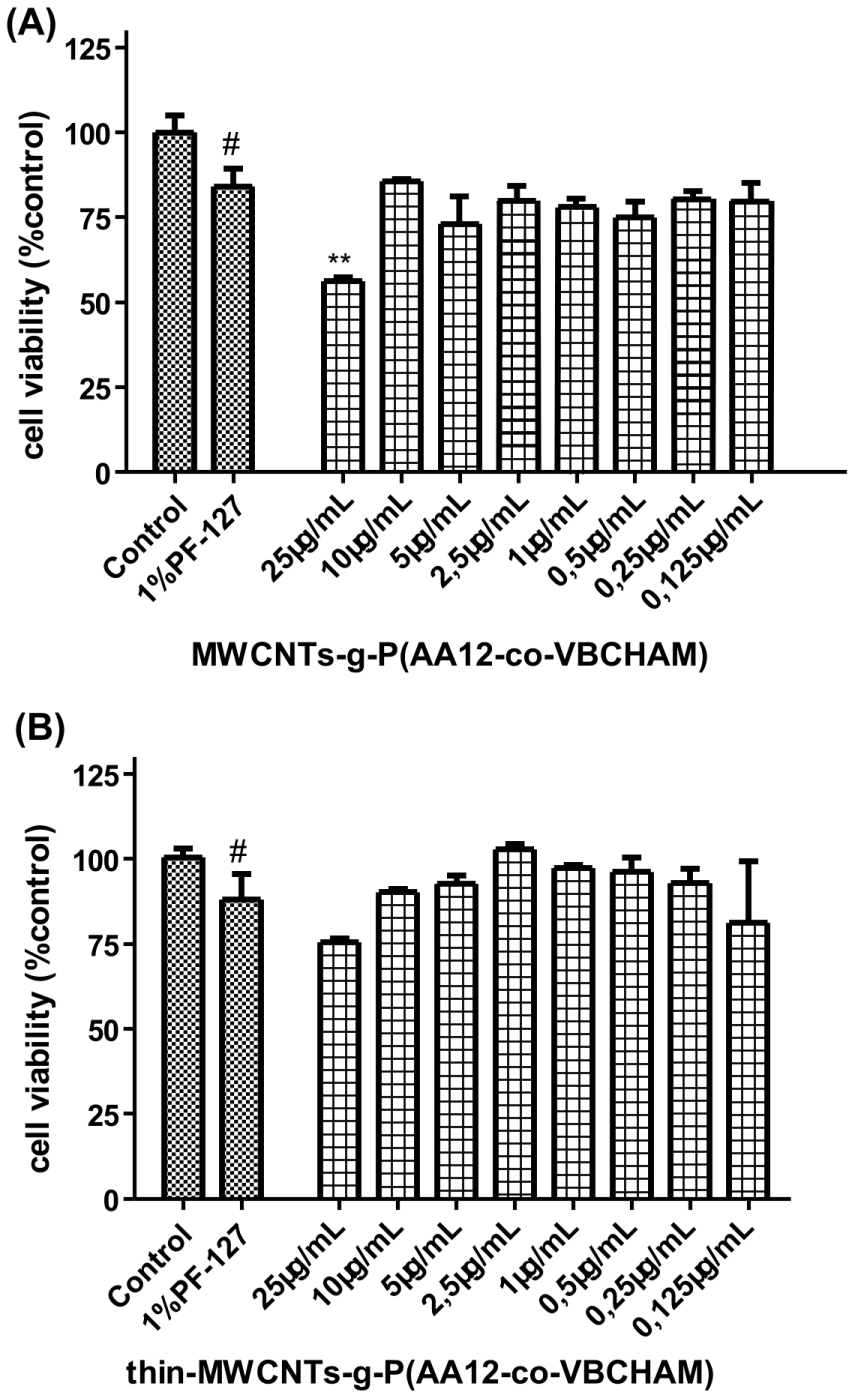
Effects of MWCNTs-g-P(AA12-co-VCHAM) on lung fibroblasts cells proliferation for a 24 h incubation period. A range of concentrations from 0.125 µg/mL to 25 µg/mL was assayed. The results are expressed as mean ±SD of three separate experiments in triplicate.

**Figure 13:**
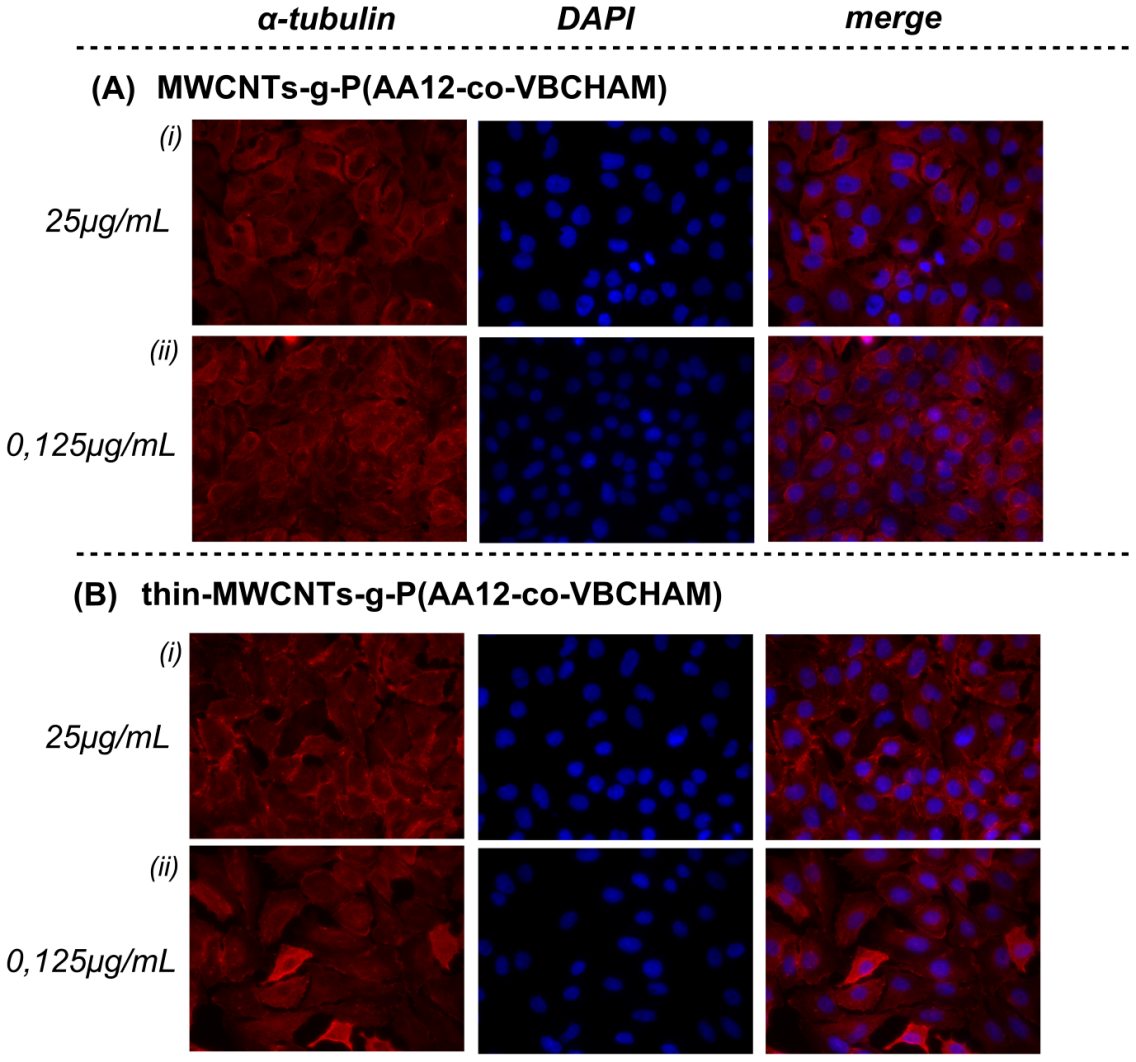
Effect of P(AA12-co-VBCHAM) decorated CNTs on lung cancer cells microtubules. Immunofluorescence staining was performed for α-tubulin (red) and nuclei (blue) in A549 cells after permeabilization. Data are representative of three independent experiments. Panel **A.** MWCNTs-g-P(AA12-co-VCHAM) (***i***) 25 µg/mL and (***ii***) 0.125 µg/mL. Panel **B.** Thin-MWCNTs-g-P(AA12-co-VCHAM) (***i***) 25 µg/mL and (***ii***) 0.125 µg/mL.

Covalent attachment of ammonium groups onto phenol-functionalized CNTs, MWCNTs-g-P(AA12-co-VCHAM), exhibited a high cell viability percentage of normal fibroblasts (70–85%) up to 10 µg/mL (**Figure 12A**). Notably, Thin-MWCNTs-g-P(AA12-co-VCHAM) exhibited no statistical significant changes in cell viability as compared to control PF-treated cells even at the highest concentration (25 µg/mL) tested. In accordance with these data, no apparent alterations in the microtubule plasticity/dynamics were observed. Microtubules extended uniformly throughout the cytoplasm and usually accumulated in the perinuclear region, where a slight increase in brightness of staining for α-tubulin was visible after treatment (**Figure 13**).

### Evaluation and comparison of functionalized CNTs toxicity

The toxicity of CNTs has been a flaming issue for biological and biomedical applications. Their growing use has raised several questions regarding their safety for human health. Research on the toxicity of CNTs at the molecular and cellular levels has been growing rapidly [52], [53]. A number of parameters including size, surface chemistry and agglomeration of nanomaterials are very crucial for the presence of cytotoxicity. Several reports in literature have shown that surface functionalization of CNTs with polymeric moieties may result in decreased toxicity levels [54]. Since CNTs are practically insoluble and hardly dispersed nanomaterials, functionalization can also improve their dispersing ability making them easier to interact with cells and facilitate the cytotoxicity studies. In the present work, the modification of CNTs with various functional groups seemed to increase cell viability. Moreover, the functionalization of CNTs with hydroxyl groups led to high cell viability even at high concentrations, comparing to pristine CNTs. Further functionalization of CNTs-OH, through the incorporation of the hydrophilic SSNa resulted in a reduction of cytotoxicity compared to the pristine CNTs, especially at low concentrations. The non-covalent interaction of PSSNa with ammonium and phosphonium groups showed more satisfactory results in the case of ammonium, as high cell viability was observed even at higher concentrations. In general, cationic compounds are promising candidates for the development of antimicrobial activity. Quaternary ammonium compounds are the most common environmentally-friendly biocidal species that can be introduced in the polymeric moieties [55]. Their biocidal activity has been previously tested through a systematic investigation with various bacterial strains [32]. In addition to this, the low toxicity profiles of CNTs containing biocidal groups through non covalent attachment (CNTs-g-PSSAmC_16_) presenting in this work, increases the importance of these materials for further investigation.

The most intriguing result, though, was the low toxicity profile reported for the CNTs-g-P(AA12-co-VBCHAM) up to 10 µg/mL. The covalent attachment of ammonium groups onto phenol-functionalized CNTs seemed to have an important effect on cell viability. It is worth mentioning that, in previous studies the vinylbenzyl dimethylhexadecylammonium chloride homopolymer (PVBCHAM) showed no biocidal activity, whereas the copolymer P(AA12-co-VBCHAM) presented high biocidal activity against various microorganisms [56]. These findings are very important and provide insights for the safe handling and use of CNTs in terms of their risk assessment to human health.

With the above-mentioned results in combination with the biocidal activity of ammonium groups [56], a further research has already been initiated in order to find the optimum conditions (polymer composition, way of attachment of the biocidal species) in order to improve cell viability.

## Conclusions

In this work, Thin-MWCNTs and MWCNTs were functionalized with antimicrobial polymers using covalent or non-covalent attachment of the biocidal species. The incorporation of biocidal groups on polymeric or monomeric moieties was accomplished through covalent or non-covalent attachment. As a result, new polymer/CNTs nanomaterials were developed and their cytotoxicity in lung cell models was studied, due to their importance in the field of Membrane Bioreactors (MBRs) for the development of new membranes of high performance. The observed toxicity was correlated with the degree of functionalization of CNTs and the structure of the functionalized groups and revealed concentration dependent cytotoxicity profiles. The results showed that the cytotoxicity of CNTs can be tuned and though significantly reduced, depending mostly on the structure of the groups incorporated on their surface.

The resulted modified CNTs nanomaterials with antimicrobial properties, either multiwalled or Thin-multiwalled, derived from the present work, will be further used in order to examine the influence of the attached groups on the dispersion efficiency as well as the embedment ability of CNTs into porous anisotropic polymeric membranes.
